# Associations of occupational stress and occupational injury with job esteem among nurses in Beijing: a cross-sectional study

**DOI:** 10.3389/fpsyg.2026.1836619

**Published:** 2026-10-21

**Authors:** Xin Wang, Lili Dong, Wenjing Zhu, Xianzong Ma, Linghui Duan, Wenyu Zhang

**Affiliations:** 1 Department of Gastroenterology, The Seventh Medical Center of Chinese PLA General Hospital, Beijing, China; 2 Pharmacy Intravenous Admixture Service, The Seventh Medical Center of Chinese PLA General Hospital, Beijing, China; 3 Department of Outpatient, The Seventh Medical Center of Chinese PLA General Hospital, Beijing, China; 4 School of Nursing, Capital Medical University, Beijing, China

**Keywords:** job esteem, nurses, occupational injury, occupational protection, occupational stress

## Abstract

**Introduction:**

Job esteem is an important aspect of occupational mental health in nurses and has been associated with multiple factors. This study aimed to explore the associations of occupational stress and occupational injury with the dimensions of job esteem among nurses in Beijing.

**Methods:**

An anonymous online survey was completed by 3,654 registered nurses in Beijing. Data were collected on demographic characteristics, occupational stress, occupational injury and job esteem. Multiple linear regression analyses were used to examine factors associated with job esteem.

**Results:**

The mean item scores for the five job esteem subscales ranged from 2.83 ± 0.69 to 3.36 ± 0.50, with professional self-awareness having the highest mean item score. The mean item score of occupational stress was 1.83 ± 0.61, with the highest mean item score observed in the nursing profession and work dimension (2.18 ± 0.70). Most participants reported at least one occupational injury or exposure during their professional career, with exposure to infectious agents being the most frequently reported category. 87.7% of participants expressed a need for occupational safety training. Occupational stress dimensions showed varying patterns of association with job esteem. Notably, higher patient nursing care scores were associated with lower scores across five job esteem dimensions, with B values ranging from −0.054 to −0.145 (all *p* ********** 0.05). Psychological harm was associated with a broader range of job esteem dimensions than physical injury. Nurses’ subjective risk perception, rather than self-reported exposure frequency in the previous year, was more closely associated with their job esteem. Perceived hospital emphasis on occupational safety was positively associated with all five job esteem subscales.

**Conclusion:**

Nurses’ job esteem was associated with occupational stress and factors related to occupational injury or exposure related factors. These findings suggest that strategies aimed at addressing occupational stress, strengthening institutional safety support, and implementing targeted psychological and physical protection measures may be promising for supporting job esteem.

## Introduction

Nurses are essential to the functioning of healthcare systems. Despite progress under China’s 14th Five-Year Plan for Developing High-Quality Healthcare Services, which aimed to expand the nursing workforce, significant challenges persist, including notably widespread job burnout, suboptimal work engagement and elevated turnover intention (Hepburn et al., 2021). Meanwhile, increasing public health demands have exacerbated these issues, intensifying the multidimensional burdens placed on nursing professionals. Previous studies have indicated that prolonged exposure to high-stress clinical environments risks may erode professional motivation and deplete emotional reserves (Wang et al., 2021). Such deterioration compromises nurses’ health, impairs clinical performance and weakens therapeutic alliances, thereby threatening overall healthcare quality and system resilience (Babapour et al., 2022).

In this context, job esteem is an important indicator of nurses’ occupational psychological well-being. Job esteem refers to the respect, value, and pride that individuals associate with their work and occupational role (Montoya and Pinter, 2024). It is particularly relevant to nursing because professional identity in nursing is closely linked to caring responsibilities, ethical commitments, and perceived social value. Social identity theory and self-determination theory suggest that individuals’ evaluations of their occupational roles can shape their self-perception, motivation, and work-related behavior (Dincer and Inangil, 2021; Ntoumanis et al., 2021; Montoya and Pinter, 2024). Higher job esteem has been associated with stronger professional agency, better clinical decision-making, and higher-quality care (Cai et al., 2020; Ghahremani et al., 2024), whereas lower job esteem has been linked to emotional exhaustion, detachment from work, and intention to leave (Liu et al., 2021a,b; Wang et al., 2023). Nurses with a strong sense of job esteem may also be better able to maintain professional values when confronted with ethical conflicts or organizational constraints (Malik et al., 2023; Lin et al., 2025).

However, this critical psychological resource may be affected by both persistent working conditions and discrete adverse events. Occupational stress represents the chronic aspect of this process. As the Healthy China 2030 strategy and healthcare reforms have increased expectations for nursing services, nurses have faced heavier workloads, greater emotional demands, additional administrative responsibilities, and continued concerns about insufficient recognition of their professional contributions. Sustained exposure to these demands can deplete psychological resources and diminish the value and pride that nurses derive from their work. Although the associations of occupational stress with burnout and turnover intention have been widely studied (Greenfield, 2020; Malik et al., 2023), less is known about whether specific occupational stressors are differentially associated with the various dimensions of job esteem.

In addition to chronic occupational stress, nurses may encounter acute work-related incidents involving biological, chemical, physical, or psychosocial hazards. In the present study, occupational injury or exposure collectively refers to work-related events that cause, or have the potential to cause, physical or psychological harm. This term is used as an umbrella concept because occupational hazards in clinical settings frequently overlap and may affect nurses through different pathways. Consistent with the broader occupational health framework established by the World Health Organization and the International Labour Organization (Greenfield, 2020; Malik et al., 2023), the concept includes four non-mutually exclusive categories: disinfectant-related exposure, exposure to infectious agents, physical injury, and psychological harm. Disinfectant-related exposure refers to occupational contact with chemical disinfectants, such as inhalation or contact with the skin or eyes, that may cause irritation or other adverse effects. Exposure to infectious agents refers to potential occupational contact with pathogens through blood, body fluids, respiratory droplets, contaminated materials, or other clinical routes; it does not necessarily indicate that an infection was subsequently diagnosed. Physical injury refers to bodily injury sustained during work, such as cuts, falls, strains, or other mechanical injuries. Psychological harm refers to emotional or psychological distress arising from work-related incidents, including verbal abuse, threats, workplace violence, bullying, or exposure to traumatic clinical events. Because a single incident may involve more than one hazard, for example, a sharp injury may entail both physical injury and potential pathogen exposure, these categories are not mutually exclusive. Research corroborates that occupational injury or exposure represents the major threat to healthcare workers, with its presentations and pathogenic mechanisms becoming notably intricate during the public health crises (Zhou et al., 2026). Epidemiological studies further report a persistently high incidence of occupational injury or exposure among healthcare personnel, with evidence supporting a clear graded association between the frequency of such exposures and subsequent adverse health outcomes (Abdelmalik et al., 2023; Zhang et al., 2025). Despite occupational injury or exposure, being recognized as a significant and acute threat, the association between occupational injury or exposure, which as a critical acute and external stressors, and the dimensions of nurses’ job esteem remain unclear and warrant in-depth investigation.

Therefore, this study aimed to describe the current levels of job esteem, and occupational stress, as well as the occurrence of occupational injury or exposure, among nurses in Beijing, China. It further examined the associations of occupational injury or exposure, conceptualized as acute and direct adversities, and occupational stress, representing chronic work-related adversity, with the core dimensions of job esteem. The findings were expected to provide a more nuanced understanding of job esteem and an empirical basis for developing targeted intervention strategies at both the organizational and societal levels.

## Methods

### Study design and setting

This cross-sectional study was conducted from January 2023 to September 2024 among registered nurses in Beijing, China. This study formed part of a broader research project examining the professional development and working conditions of the nursing workforce in Beijing. The study protocol, including the questionnaire structure and methodology, underwent three rounds of rigorous expert review by specialists from the Chinese PLA General Hospital, Peking Union Medical College, and Capital Medical University to ensure content validity.

Nurses were eligible to participate if they met the following criteria: (1) held a valid Chinese nursing license, (2) were currently employed in a clinical nursing role, (3) had at least 2 years of full-time clinical nursing experience, and (4) agreed voluntarily to participate. Nurses working exclusively in administrative or other non-clinical positions were not eligible. Nurses who self-reported a diagnosed psychiatric disorder or severe chronic condition that could substantially interfere with their ability to work or questionnaire completion were also excluded. This study was conducted in accordance with the Declaration of Helsinki and was approved by the Medical Ethics Committee of Capital Medical University (Approval No. Z2023SY051).

### Data collection

To ensure a representative sample of the nursing workforce in Beijing, a stratified cluster sampling design was implemented. First, hospitals in Beijing were stratified based on three key criteria: hospital grade (tertiary, secondary, or primary), geographic location (urban, suburban, or exurban), and institutional ownership (public or private). Within each stratum, purposive sampling was applied to select hospitals deemed representative of the stratum characteristics, yielding a total of 14 participating hospitals. Within each selected hospital, a census sampling method was employed. Rather than selecting a subset of nurses, all registered nurses listed in the personnel registries of the participating hospitals who met the eligibility criteria were invited to participate. This approach eliminated selection bias at the individual level within the participating units.

The required sample size was calculated using the formula: *N* = (Uασ/δ). Based on a standard deviation (*σ*) of 1.15, a significance level (*α*) of 0.05 (corresponding to Zα = 1.96 for a two-tailed test), and a precision level (δ) of 0.066, the initial minimum sample size was determined to be 1,167. Allowing for a potential 10% nonresponse or invalid-response rate, the recruitment target was adjusted to 1,297. To maximize statistical power and representativeness, all eligible nurses (3,654) meeting the inclusion criteria were invited to participate.

Data collection was conducted using the SO JUMP platform.^1^ An anonymous survey link, configured to allow only one submission per respondent, was distributed to all eligible nurses via institutional communication channels and professional networks. Prior to participation, all individuals received information about the study’s purpose and procedures and provided electronic informed consent, which they were required to confirm before starting the survey. Participation was entirely voluntary, and respondents could withdraw at any time without providing a reason.

### Questionnaire development

The survey comprised four parts.

Part one collected demographic information, including gender, age, employment type, education level, average monthly income, and professional title.

Part two assessed occupational injury or exposure using the following items: Have you ever experienced an occupational injury or exposure during their professional career? (yes or no); Type of occupational injury or exposure (disinfectant-related exposure; psychological harm; exposure to infectious agents; physical injury); How frequently have you been exposed to occupational injuries (times in recent 1 year)? (0, 1–5, 6–10, 11–15, ≥ 16); Perceived risk of occupational exposure (none; low; moderate; high; very high); Do you need occupational safety and protection training? (yes or no); Perceived hospital emphasis on occupational safety (none; low; moderate; high; very high).

Part three assessed job esteem using the validated Chinese version of the job esteem scale (JES). The scale was originally constructed by Choi and Jung (2020), and later translated by Shi and Shen (2021). Its subsequent use in Chinese nursing populations includes the study by Guo et al. (2026), which reported a Cronbach’s α of 0.970. The Chinese JES consists of 25 items across five dimensions: professional self-awareness (4 items), social trust and respect (3 items), organizational respect and recognition (4 items), professional authority and future value (4 items), and professional competence (10 items). Each item was rated on a 5-point Likert scale, ranging from 1 (completely disagree) to 5 (completely agree). Higher scores indicate greater job esteem. In the present study, Cronbach’s α was 0.921 for the total scale. The Cronbach’s α coefficients for the five dimensions were 0.890 for professional self-awareness, 0.888 for social trust and respect, 0.877 for organizational respect and recognition, 0.940 for professional authority and future value, and 0.960 for professional competence.

Part four was used to assess occupational stress using the scale developed by Li and Liu (2000), which was made and revised based on the nurse occupational stress scale from the United States and the United Kingdom, according to China’s national conditions, and is currently the most used scale for investigation on nurse occupational stress in China. It comprises 35 items across five dimensions: nursing profession and work (7 items), workload and time allocation (5 items), work environment and resources (3 items), patient nursing care (11 items), and nursing management and interpersonal relationships (9 items). Items are rated on a 4-point Likert scale ranging from 1 (mild) to 4 (severe). The scale demonstrated excellent internal consistency (Cronbach’s *α* = 0.980).

### Data management and quality control

Given the use of an online survey platform, procedural controls were implemented to ensure data integrity. The survey was configured with mandatory fields to prevent missing data at the source. Following data collection, raw data were exported directly from the SO JUMP platform into Microsoft Excel, eliminating manual entry errors. A rigorous data cleaning protocol was applied to identify invalid submissions based on two criteria: (1) a completion time of less than 120 s, suggesting insufficient attention to item content; and (2) discernible response patterns (e.g., straight-lining or identical answers for all questions). These invalid responses were excluded from the final dataset prior to statistical analysis.

Several procedural measures were incorporated into the study design to mitigate common method bias, including ensuring respondent anonymity to reduce social desirability bias and structurally separating predictor and outcome variables within the questionnaire. Additionally, Harman’s single-factor test was conducted using exploratory factor analysis, revealing that the first factor accounted for 26.2% of the total variance (well below the critical threshold of 40%). Harman’s single-factor test suggested that common method variance was unlikely to be dominated by a single factor; however, common method bias could not be completely excluded.

### Statistical analysis

All statistical analyses were performed using SPSS version 23.0 (IBM Corp., Armonk, NY, USA). Continuous variables were summarized as means ± standard deviations (*M* ± SD), and categorical variables as frequencies (percentages). Pearson correlations were used to assess bivariate associations among continuous variables. Five multiple linear regression models were fitted separately, with scores for professional self-awareness, social trust and respect, professional competence, organizational respect and recognition, and professional authority and future value as continuous outcomes. Each model included 20 predictors covering demographic characteristics, occupational injury or exposure, and occupational stress. Categorical predictors with more than two categories were dummy-coded using prespecified reference groups. Adjacent categories with small cell sizes were combined before analysis and the resulting groupings were used consistently across all models. Binary predictors were entered directly, with 0-coded categories as references, and mean item scores for the five occupational stress dimensions were treated as continuous predictors. Multicollinearity was assessed using variance inflation factors (VIFs). Residual histograms, Q–Q plots, and residual plots were examined to assess normality, linearity, and homoscedasticity. Potential hospital-level clustering was evaluated using intraclass correlation coefficients (ICCs) calculated from empty multilevel models for each outcome. Associations were reported as unstandardized regression coefficients (*B*) with 95% confidence intervals (CIs) and *p* values. A two-sided *p* < 0.05 was considered statistically significant.

## Results

### Demographic characteristics of the participants

The sample included 3,654 nurses, of whom 3,482 (95.3%) were female and 172 (4.7%) were male. Most participants were aged ≤35 years (2,547, 69.7%), followed by those aged 36–50 years (982, 26.9%) and ≥51 years (125, 3.4%). Regarding employment status, most participants were employed as contract nurses (2,339, 64.0%). Most participants held a bachelor’s degree (2,804, 76.7%), followed by those with higher vocational education (763, 20.9%). More than two-thirds reported a monthly income of ≤RMB 10,000 (2,518, 68.9%). Regarding professional titles, the largest category was nurse practitioners (1,550, 42.4%), followed by nurses-in-charge (1,121, 30.7%). Participants with senior professional titles represented a small proportion of the sample. The demographic characteristics of the participants are shown in Table 1.

**Table 1:** Demographic characteristics of nurses in Beijing (*N* = 3,654).

Variables	Frequency, *n* (*%*)
Gender	
Male	172 (4.7)
Female	3,482 (95.3)
Age	
≤ 35	2,547 (69.7)
36–50	982 (26.9)
≥ 51	125 (3.4)
Employment type	
Permanent nurse	994 (27.2)
Contract nurse	2,339 (64.0)
Others	321 (8.8)
Education	
Secondary vocational education	39 (1.1)
Higher vocational education	763 (20.9)
Bachelor’s degree	2,804 (76.7)
Master’s degree	48 (1.3)
Income per month, RMB	
≤ 10,000	2,518 (68.9)
≥ 10,001	1,136 (31.1)
Professional titles	
Nurse	880 (24.1)
Nurse Practitioner	1,550 (42.4)
Nurse-in-charge	1,121 (30.7)
Vice Professor	92 (2.5)
Chief nurse	11 (0.3)

### Characteristics of job esteem, occupational stress, and occupational injury or exposure

A total of 3,259 nurses (89.2%) reported having ever experienced an occupational injury or exposure during their professional career. Exposure to infectious agents was reported by 2,641 participants (72.3%), psychological harm by 1,584 (43.3%), disinfectant-related exposure by 1,483 (40.6%), and physical injury by 1,120 (30.7%). Regarding occupational injury or exposure in the previous year, 1,046 participants (28.6%) reported no occupational injury or exposure. 2,325 (63.6%) reported 1–5 events, 139 (3.8%) reported 6–10 events, 30 (0.8%) reported 11–15 events, and 114 (3.1%) reported ≥16 events. Perceived occupational exposure risk was rated as none by 464 participants (12.7%), low by 1,062 (29.1%), moderate by 1,253 (34.3%), high by 682 (18.7%), and very high by 193 (5.3%). A total of 3,203 nurses (87.7%) reported a need for occupational safety and protection training. Perceived hospital emphasis on occupational safety was rated as none by 72 participants (2.0%), low by 322 (8.8%), moderate by 955 (26.1%), high by 1,445 (39.5%), and very high by 860 (23.5%). Characteristics related to job esteem, occupational stress, and occupational injury or exposure, are shown in Table 2.

**Table 2:** Occupational injury or exposure characteristics among nurses (*N* = 3,654).

Variables	Frequency, *n* (*%*)
Have you ever experienced an occupational injury or exposure during their professional career?	
Yes	3,259 (89.2)
No	395 (10.8)
Type of occupational injury or exposure	
Disinfectant-related exposure	1,483 (40.6)
Psychological harm	1,584 (43.3)
Exposure to infectious agents	2,641 (72.3)
Physical injury	1,120 (30.7)
How frequently have you been exposed to occupational injuries (times in recent 1 year)?	
0	1,046 (28.6)
1–5	2,325 (63.6)
6–10	139 (3.8)
11–15	30 (0.8)
≥ 16	114 (3.1)
Perceived risk of occupational exposure	
None	464 (12.7)
Low	1,062 (29.1)
Moderate	1,253 (34.3)
High	682 (18.7)
Very high	193 (5.3)
Do you need occupational safety and protection training?	
Yes	3,203 (87.7)
No	451 (12.3)
Perceived Hospital Emphasis on Occupational Safety	
None	72 (2.0)
Low	322 (8.8)
Moderate	955 (26.1)
High	1,445 (39.5)
Very high	860 (23.5)

Type of occupational injury or exposure: multiple responses were allowed; percentages were calculated using the total sample (*N* = 3,654) as the denominator.

The scores (*M* ± *SD*) for the five job esteem subscales were as follows: professional self-awareness, 13.44 ± 2.00; social trust and respect, 10.04 ± 1.63; organizational respect and recognition, 12.49 ± 2.40; professional authority and future value, 11.32 ± 2.74; and professional competence, 32.59 ± 4.77. The corresponding mean item scores for the subscales were 3.36 ± 0.50, 3.35 ± 0.54, 3.12 ± 0.60, 2.83 ± 0.69, and 3.26 ± 0.48, respectively. Meanwhile, the mean overall occupational stress score was 64.19 ± 21.20, corresponding to a mean item score of 1.83 ± 0.61. Nursing profession and work had the highest mean item score (2.18 ± 0.70), followed by workload and time allocation (2.13 ± 0.83), whereas nursing management and interpersonal relationships had the lowest mean item score (1.52 ± 0.63) (Table 3).

**Table 3:** Characteristics of job esteem and occupational stress (*N* = 3,654).

Dimension	Total score, *mean ± SD*	Mean item score, *mean ± SD*
Job esteem	79.90 ± 11.47	3.20 ± 0.46
Professional self-awareness	13.44 ± 2.00	3.36 ± 0.50
Social trust and respect	10.04 ± 1.63	3.35 ± 0.54
Organizational respect and recognition	12.49 ± 2.40	3.12 ± 0.60
Professional authority and future value	11.32 ± 2.74	2.83 ± 0.69
Professional competence	32.59 ± 4.77	3.26 ± 0.48
Occupational Stress	64.19 ± 21.20	1.83 ± 0.61
Nursing profession and work	15.24 ± 4.93	2.18 ± 0.70
Workload and time allocation	10.65 ± 4.14	2.13 ± 0.83
Work environment and resources	4.93 ± 2.26	1.64 ± 0.75
Patient nursing care	19.68 ± 7.43	1.79 ± 0.68
Nursing management and interpersonal relationships	13.70 ± 5.63	1.52 ± 0.63

### Correlations between job esteem and occupational stress

Pearson correlation analyses showed that all five occupational stress dimensions were significantly and negatively correlated with all five job esteem dimensions, with correlation coefficients ranging from *r* = −0.375 to −0.098 (all *p* < 0.001). Professional authority and future value showed the strongest negative correlations with workload and time allocation (*r* = −0.375), nursing profession and work (*r* = −0.346), and patient nursing care (*r* = −0.297). The correlations between job esteem and occupational stress are shown in Table 4.

**Table 4:** Pearson correlations between job esteem and occupational stress scale (*N* = 3,654).

Job esteem dimension	Nursing profession and work	Workload and time allocation	Work environment and resources	Patient nursing care	Nursing management and interpersonal relationships
Professional self-awareness	−0.159	−0.189	−0.160	−0.200	−0.196
Social trust and respect	−0.109	−0.120	−0.098	−0.125	−0.137
Organizational respect and recognition	−0.290	−0.316	−0.191	−0.251	−0.229
Professional authority and future value	−0.346	−0.375	−0.203	−0.297	−0.240
Professional competence	−0.141	−0.170	−0.113	−0.164	−0.152

Values are Pearson correlation coefficients (r). All correlations were statistically significant at *p* < 0.01 (two-sided).

### Multiple linear regression analyses of factors associated with job esteem dimensions

Across the five regression models, VIFs ranged from 1.014 to 3.409, indicating no substantial multicollinearity. Visual inspection of residual histograms, Q-Q plots, and residual plots suggested no major departures from normality, linearity, or homoscedasticity. Hospital-level ICCs ranged from 0.29 to 1.46%, suggesting limited clustering.

Employment type was significantly associated with four job esteem dimensions. Compared with permanent nurses, contract nurses scored significantly higher on professional self-awareness, social trust and respect, professional competence, and professional authority and future value. Monthly income was positively associated with professional self-awareness, social trust and respect, and professional competence. Age was significantly associated only with professional authority and future value. Compared with nurses aged ≤35 years, those aged 36–50 years and ≥51 years had significantly higher scores on professional authority and future value, indicating an increasing trend with age. Professional title was significantly associated only with professional authority and future value. Compared with nurses, nurse practitioners and nurse**–**in**–**charge had significantly lower scores on professional authority and future value.

Psychological harm was negatively associated with professional self**–**awareness, professional competence, organizational respect and recognition, and professional authority and future value. Physical injury was negatively associated with professional competence, organizational respect and recognition, and professional authority and future value. Disinfectant-related exposure was positively associated with social trust and respect, organizational respect and recognition, and professional authority and future value. The frequency of occupational injury or exposure was significantly associated only with social trust and respect. Compared with reporting no occupational injury or exposure events, reporting 6–10 or 11–15 events were associated with lower scores on social trust and respect.

The reported need for occupational safety and protection training was positively associated with all five job esteem dimensions. Compared with nurses who perceived no risk, those perceiving low, moderate, and high risk had significantly lower scores on professional self-awareness, social trust and respect, professional competence, organizational respect and recognition, and professional authority and future value. Notably, for organizational respect and recognition and professional authority and future value, higher perceived risk was associated with progressively lower scores.

Perceived hospital emphasis on occupational safety demonstrated the strongest positive associations across all five job esteem dimensions. Compared with nurses who reported no or low perceived emphasis, those who perceived high emphasis had significantly higher scores on professional self-awareness, social trust and respect, professional competence, organizational respect and recognition, and professional authority and future value. Similarly, nurses who perceived very high hospital emphasis on occupational safety had higher scores across all five dimensions than those in the reference group.

Occupational stress dimensions demonstrated varying patterns of association with job esteem. Higher workload and time allocation scores were associated with lower scores on four of the five job esteem dimensions: professional self-awareness, professional competence, organizational respect and recognition, and professional authority and future value. Similarly, scores in the patient nursing care were negatively associated with all five job esteem dimensions, indicating that greater nursing care burden was uniformly associated with lower scores across all aspects of job esteem. The nursing profession and work score was negatively associated with organizational respect and recognition, and professional authority and future value. In contrast, the work environment and resources score was positively associated with professional competence, organizational respect and recognition, and professional authority and future value. The nursing management and interpersonal relationships score was positively associated only with professional authority and future value (Table 5).

**Table 5:** Multiple linear regression analysis of factors associated with job esteem among nurses in Beijing (*N* = 3,654).

Variables	Professional self-awareness		Social trust and respect		Professional competence		Organizational respect and recognition		Professional authority and future value	
	*B* (95% CI)	*p* value	*B* (95% CI)	*p* value	*B* (95% CI)	*p* value	*B* (95% CI)	*p* value	*B* (95% CI)	*p* value
Gender										
Female	Reference		Reference		Reference		Reference		Reference	
Male	−0.058 (−0.130, 0.014)	0.115	−0.057 (−0.137, 0.023)	0.163	−0.043 (−0.113, 0.026)	0.218	0.015 (−0.068, 0.099)	0.721	−0.026 (−0.119, 0.067)	0.590
Age										
≤35 years	Reference		Reference		Reference		Reference		Reference	
36–50 years	0.039 (−0.005, 0.084)	0.084	0.049 (−0.001, 0.098)	0.054	0.035 (−0.008, 0.078)	0.108	−0.028 (−0.080, 0.024)	0.292	0.079 (0.021, 0.137)	0.007
≥51 years	0.042 (−0.053, 0.137)	0.386	0.019 (−0.086, 0.124)	0.726	0.062 (−0.029, 0.154)	0.180	−0.099 (−0.209, 0.010)	0.076	0.201 (0.078, 0.323)	0.001
Employment type										
Permanent nurse	Reference		Reference		Reference		Reference		Reference	
Contract nurse	0.069 (0.007, 0.130)	0.028	0.100 (0.032, 0.168)	0.004	0.089 (0.030, 0.148)	0.003	0.060 (−0.011, 0.131)	0.100	0.086 (0.007, 0.166)	0.033
Others	0.048 (−0.006, 0.102)	0.079	0.079 (0.019, 0.139)	0.010	0.082 (0.030, 0.134)	0.002	0.028 (−0.035, 0.091)	0.382	0.030 (−0.040, 0.100)	0.339
Educational level										
Secondary vocational and Higher vocational	Reference		Reference		Reference		Reference		Reference	
Bachelor’s degree	−0.001 (−0.040, 0.038)	0.972	−0.010 (−0.053, 0.034)	0.661	−0.008 (−0.046, 0.029)	0.663	−0.027 (−0.072, 0.018)	0.240	−0.044 (−0.095, 0.006)	0.085
Master’s _degree	−0.054 (−0.195, 0.087)	0.456	0.074 (−0.083, 0.230)	0.357	0.047 (−0.088, 0.183)	0.494	−0.009 (−0.172, 0.155)	0.919	−0.022 (−0.204, 0.161)	0.816
Monthly income										
≤10,000	Reference		Reference		Reference		Reference		Reference	
≥10,001	0.058 (0.023, 0.092)	0.001	0.060 (0.021, 0.098)	0.002	0.048 (0.015, 0.082)	0.005	0.024 (−0.017, 0.064)	0.251	0.038 (−0.007, 0.083)	0.094
Professional title										
Nurse	Reference		Reference		Reference		Reference		Reference	
Nurse practitioner	−0.003 (−0.044, 0.038)	0.872	−0.003 (−0.048, 0.042)	0.897	−0.003 (−0.043, 0.036)	0.863	−0.024 (−0.071, 0.024)	0.331	−0.064 (−0.117, −0.011)	0.019
Nurse-in-charge	0.024 (−0.029, 0.077)	0.379	0.030 (−0.029, 0.089)	0.315	0.021 (−0.030, 0.072)	0.419	−0.024 (−0.086, 0.037)	0.438	−0.091 (−0.160, −0.022)	0.009
Vice/Chief nurse	0.065 (−0.050, 0.181)	0.268	0.112 (−0.017, 0.240)	0.089	0.024 (−0.087, 0.136)	0.668	−0.025 (−0.159, 0.110)	0.720	0.028 (−0.121, 0.178)	0.710
Have you ever experienced an occupational injury or exposure?										
No	Reference		Reference		Reference		Reference		Reference	
Yes	−0.037 (−0.089, 0.016)	0.176	−0.028 (−0.087, 0.031)	0.354	−0.044 (−0.095, 0.007)	0.093	−0.014 (−0.075, 0.048)	0.665	−0.018 (−0.086, 0.051)	0.609
Disinfectant-related exposure										
No	Reference		Reference		Reference		Reference		Reference	
Yes	0.020 (−0.015, 0.054)	0.260	0.039 (0.001, 0.077)	0.043	0.022 (−0.010, 0.055)	0.181	0.040 (0.001, 0.080)	0.046	0.063 (0.019, 0.107)	0.005
Psychological harm										
No	Reference		Reference		Reference		Reference		Reference	
Yes	−0.053 (−0.088, −0.019)	0.002	−0.037 (−0.075, 0.001)	0.058	−0.039 (−0.071, −0.006)	0.022	−0.079 (−0.119, −0.039)	<0.001	−0.113 (−0.158, −0.069)	<0.001
Exposure to infectious agents										
No	Reference		Reference		Reference		Reference		Reference	
Yes	0.017 (−0.024, 0.058)	0.420	0.005 (−0.041, 0.050)	0.844	0.006 (−0.033, 0.046)	0.755	0.001 (−0.046, 0.049)	0.953	−0.021 (−0.074, 0.032)	0.434
Physical injury										
No	Reference		Reference		Reference		Reference		Reference	
Yes	−0.025 (−0.061, 0.011)	0.178	−0.017 (−0.057, 0.023)	0.400	−0.037 (−0.071, −0.002)	0.040	−0.067 (−0.109, −0.025)	0.002	−0.088 (−0.134, −0.041)	<0.001
Do you need occupational safety and protection training?										
No	Reference		Reference		Reference		Reference		Reference	
Yes	0.059 (0.013, 0.104)	0.012	0.062 (0.011, 0.113)	0.018	0.055 (0.011, 0.099)	0.014	0.117 (0.064, 0.170)	<0.001	0.132 (0.073, 0.191)	<0.001
Frequency of occupational injury exposure										
None	Reference		Reference		Reference		Reference		Reference	
1–5 times	−0.017 (−0.054, 0.020)	0.371	−0.026 (−0.067, 0.015)	0.212	−0.015 (−0.050, 0.021)	0.421	−0.039 (−0.081, 0.004)	0.075	0.084 (−0.043, 0.211)	0.197
6–10	−0.063 (−0.152, 0.027)	0.169	−0.184 (−0.283, −0.086)	0.001	−0.065 (−0.151, 0.021)	0.137	−0.056 (−0.162, 0.050)	0.300	−0.014 (−0.134,0.107)	0.823
11–15 times	−0.017 (−0.101, 0.068)	0.697	−0.254 (−0.382, −0.126)	<0.001	−0.026 (−0.108, 0.055)	0.524	−0.052 (−0.150, 0.047)	0.303	−0.017 (−0.173, 0.139)	0.830
≥16 times	−0.070 (−0.239, 0.098)	0.415	−0.070 (−0.258, 0.117)	0.461	−0.045 (−0.207, 0.117)	0.588	0.013 (−0.183, 0.208)	0.897	0.062 (−0.191, 0.314)	0.632
Perceived occupational risk										
None	Reference		Reference		Reference		Reference		Reference	
Low	−0.076 (−0.128, −0.023)	0.005	−0.075 (−0.133, −0.017)	0.012	−0.102 (−0.153, −0.052)	<0.001	−0.109 (−0.170, −0.048)	<0.001	−0.084 (−0.152, −0.016)	0.015
Moderate	−0.092 (−0.145, −0.039)	0.001	−0.105 (−0.164, −0.046)	0.001	−0.129 (−0.180, −0.077)	<0.001	−0.164 (−0.225, −0.102)	<0.001	−0.150 (−0.218, −0.081)	<0.001
High	−0.086 (−0.145, −0.027)	0.004	−0.067 (−0.133, −0.002)	0.044	−0.113 (−0.170, −0.056)	<0.001	−0.169 (−0.237, −0.100)	<0.001	−0.183 (−0.259, −0.106)	<0.001
Very high	−0.067 (−0.149, 0.014)	0.106	0.037 (−0.053, 0.127)	0.423	−0.044 (−0.122, 0.035)	0.274	−0.188 (−0.282, −0.093)	<0.001	−0.207 (−0.312, −0.101)	<0.001
Perceived hospital emphasis on occupational safety										
None and low	Reference		Reference		Reference		Reference		Reference	
Moderate	−0.006 (−0.061, 0.049)	0.827	0.058 (−0.003, 0.118)	0.063	0.006 (−0.047, 0.058)	0.837	0.027 (−0.036, 0.090)	0.405	−0.027 (−0.098, 0.044)	0.455
High	0.138 (0.085, 0.190)	<0.001	0.149 (0.091, 0.208)	<0.001	0.114 (0.063, 0.165)	<0.001	0.169 (0.108, 0.230)	<0.001	0.137 (0.069, 0.205)	<0.001
Very high	0.360 (0.303, 0.417)	<0.001	0.408 (0.344, 0.471)	<0.001	0.346 (0.291, 0.401)	<0.001	0.438 (0.372, 0.504)	<0.001	0.399 (0.325, 0.473)	<0.001
Occupational stress dimensions										
Nursing profession and work score	0.036 (−0.001, 0.072)	0.054	0.008 (−0.033, 0.048)	0.704	0.021 (−0.014, 0.056)	0.234	−0.061 (−0.103, −0.018)	0.005	−0.096 (−0.143, −0.049)	<0.001
Nursing management and interpersonal relationships score	−0.030 (−0.071, 0.011)	0.154	−0.041 (−0.086, 0.005)	0.081	−0.010 (−0.050, 0.029)	0.616	−0.010 (−0.058, 0.037)	0.675	0.061 (0.008, 0.114)	0.025
Workload and time allocation score	−0.038 (−0.070, −0.005)	0.024	−0.013 (−0.049, 0.024)	0.497	−0.044 (−0.075, −0.012)	0.007	−0.111 (−0.149, −0.073)	<0.001	−0.179 (−0.222, −0.137)	<0.001
Patient nursing care score	−0.091 (−0.132, −0.050)	<0.001	−0.054 (−0.099, −0.008)	0.021	−0.084 (−0.123, −0.045)	<0.001	−0.078 (−0.125, −0.031)	0.001	−0.145 (−0.198, −0.092)	<0.001
Work environment and resources score	0.016 (−0.014, 0.046)	0.286	0.028 (−0.006, 0.061)	0.102	0.043 (0.014, 0.072)	0.004	0.069 (0.034, 0.103)	<0.001	0.111 (0.072, 0.150)	<0.001

Job esteem and occupational stress dimensions were entered as mean item scores. B, unstandardized regression coefficient; CI, confidence interval. The following categories were collapsed prior to analysis: (1) educational level: secondary vocational education and higher vocational education were combined as the reference category; (2) professional title: vice chief nurse and chief nurse were combined into vice/chief nurse; (3) perceived hospital emphasis on occupational safety: none and low were combined as the reference category (none and low).

## Discussion

The demographic findings of this study portray a nursing workforce that is predominantly young, female, and educated to the bachelor’s degree level (see Table 1). The overwhelming predominance of female participants is consistent with the global nursing workforce, which remains a female-dominated profession (Zhang et al., 2022; Guo et al., 2026). Another notable finding is the high educational level, which substantially exceeds that reported in many other national contexts (Zhang et al., 2021; see Table 1). This phenomenon may reflect the continuing development of degree-level nursing education in China, and it may also be partly attributable to the location of the study in Beijing, where higher education and tertiary healthcare resources are highly concentrated. The distribution of professional titles showed a typical pyramidal structure with a broad base of nurses and nurse practitioners, and an extremely narrow apex of nurses with senior leadership (vice professor and chief nurse). While this structure is typical in highly hierarchical professions, it highlights the intense competition for limited senior positions. The scarcity of advanced roles may be related to fewer career advancement opportunities and limited access to mentorship for younger nurses, and these factors may be associated with professional development and job satisfaction.

Among the five dimensions of job esteem, professional self–awareness had the highest mean item score, although it was only marginally higher than social trust and respect (see Table 3). This finding suggests that nurses generally reported a relatively positive perception of their professional identity and personal value within the profession. In contrast, professional authority and future value received the lowest score, suggesting less favorable perceptions of the social status and future prospects of nursing. This finding is consistent with previous studies, which have suggested that many nurses in China perceive nursing as receiving insufficient societal recognition and inadequate respect from patients and the public. The mean item score for organizational respect and recognition was also lower than those for professional self–awareness and social trust and respect, suggesting that institutional recognition, professional support, and acknowledgment of nurses’ contributions may be relevant to job esteem (Karanikola et al., 2018).

Meanwhile, nurses’ mean occupational stress scores varied across dimensions. Among all the subscales, nursing profession and work and workload and time allocation had the highest mean item scores, suggesting that profession-related concerns, heavy workloads, and time constraints may be prominent stressors (see Table 3). This pattern is consistent with previous studies identifying high workload, insufficient staffing, time pressure, and emotionally demanding patient care as major stressors among nurses (Liu et al., 2021a,b; Brassington et al., 2025). Research conducted in other countries has similarly reported associations between high workload, staffing constraints and burnout, reduced job satisfaction, and occupational injury risk (Liu et al., 2021a,b). These findings suggest that workload may be a shared challenge across healthcare systems, although differences in healthcare organization and staffing standards should be considered when making international comparisons. Notably, in our study, the dimension of nursing management and interpersonal relationships had the lowest mean item score, suggesting that issues related to management and relationship were perceived as less stressful than the other occupational stress domains. This finding differs from that reported by Zhang et al. (2023), who found that the majority of nurses perceived inadequate respect from clinical managers and physicians. The discrepancy may reflect differences in study populations, hospital characteristics, measurement instruments, or nursing management practices.

Regarding occupational injury or exposure, the majority of nurses reported having experienced at least one occupational injury or exposure during their professional career. Exposure to infectious agents was the most frequently reported type, followed by psychological harm (see Table 2). These findings are broadly consistent with international evidence, suggesting that nurses frequently experience biological exposure, sharps injuries, musculoskeletal injuries, workplace violence, and psychological harm (GBD 2023 Disease and Injury and Risk Factor Collaborators, 2025; Sanjobo et al., 2025). Although direct comparisons of prevalence across studies are difficult because of a series of factors (Soroush et al., 2018; GBD 2023 Disease and Injury and Risk Factor Collaborators, 2025; Sanjobo et al., 2025), the consistently high burden reported across countries underscores the importance of occupational health and safety among nurses. Notably, although most participants perceived their hospitals’ emphasis on occupational safety as high or very high, the majority of nurses still reported a need for occupational safety and protection training (see Table 2). These findings suggest that favorable perceptions of organizational attention to safety may coexist with a perceived need for ongoing training, skills updating, and risk-prevention education. This observation is broadly compatible with safety climate theory, which proposes that employees’ perceptions of organizational commitment to safety may be associated with safety–related attitudes and behaviors (Loh et al., 2025).

This study also investigated the multiple factors associated with job esteem. Nurses with higher monthly income reported higher levels of job esteem (see Table 5). This finding is consistent with previous research, which showed that salary level, as a direct reflection of an employee’s contribution to the organization, is associated with nurses’ sense of professional respect (Zhang et al., 2023). From the perspective of social exchange theory, fair compensation may signal that an organization values employees’ work, which may be associated with more favorable professional evaluations. Maslow’s hierarchy of needs also suggests that economic security may be associated with greater attention to higher-order needs, including recognition and esteem (Loh et al., 2025).

The findings also indicated that each dimension of occupational stress was significantly correlated with job esteem dimensions (see Tables 4, 5). Patient nursing care was the only stress dimension that was negatively associated with all five job esteem dimensions. This dimension may reflect the emotional, cognitive, and practical demands involved in direct patient care, including responsibility for patient outcomes, communication with patients and families, and exposure to suffering or conflict. Such demands may be relevant to how nurses evaluate their professional competence, social recognition, organizational value, and career prospects. Workload and time allocation was negatively associated with professional self-awareness, professional competence, organizational respect and recognition, and professional authority and future value, and the strongest correlation was observed between workload and time allocation and professional authority and future value. One possible interpretation, consistent with the Job Demands-Resources model, is that sustained workload and time pressure may be associated with fewer available resources for professional development, reflective practice, and long-term career planning (Almino et al., 2025; Portoghese et al., 2025). Nurses facing persistent time pressure may also perceive fewer opportunities to obtain advanced qualifications or engage in professional activities, which may be associated with less favorable expectations regarding the future of their careers (Chao and Liao, 2025). Nursing profession and work was negatively associated with organizational respect and recognition and professional authority and future value. These associations suggest that stress related to the nature, responsibilities, and social position of nursing may be relevant to nurses’ perceptions of organizational recognition and long-term professional prospects.

Furthermore, this study found that psychological harm showed associations with a broader range of job esteem dimensions than physical injury, and psychological harm showed the strongest association with professional authority and future value (see Table 5). This differential pattern is noteworthy and merits further exploration. Previous research has indicated that psychological harm may be associated with nurses’ core professional identity and may undermine their sense of professional competence and belonging (Cai et al., 2020; Liu et al., 2021a,b). Moreover, psychological harm has been proposed to be cumulative and persistent in daily work, and its association with job esteem may be stronger than that of physical injuries, which are often episodic (Wang et al., 2023). Nursing, which is characterized by high emotional labor, involves prolonged exposure to patient suffering, end-of-life scenarios, and patient–nurse conflicts (Malik et al., 2023; Zhang et al., 2023); the cumulative burden of psychological harm may be associated with nurses’ overall perceptions of themselves, their organization, and their professional future. Previous studies have similarly suggested that psychological violence is more strongly associated with nurse burnout and turnover intention than physical violence (Liu et al., 2018).

Additionally, the positive association between disinfectant exposure and job esteem is somewhat unexpected and may reflect unmeasured confounding by job role (see Table 5). Nurses with frequent disinfectant exposure may work in specialized units such as operating rooms, ICUs, or sterile supply centers, which may offer higher technical recognition, clearer career pathways, and more comprehensive protective measures (Wang et al., 2025). Another possible explanation is that nurses working in environments with high disinfectant exposure may have a stronger sense of professional identity associated with specialized skills and team cohesion. This finding should be interpreted with caution and warrants further investigation. Notably, the need for occupational safety training was positively associated with all five job esteem dimensions. Although this association may appear counterintuitive, it may indicate that nurses who report safety training needs have greater safety awareness and professional engagement, which may be positively associated with their professional self-evaluation. Additionally, nurses who perceive that their organizations address these training needs, may also perceive greater organizational support, which may be associated with higher job esteem.

Perceived risk of occupational exposure was associated with professional self–awareness, professional competence, organizational respect and recognition, and professional authority and future value, whereas self-reported frequency of exposure was only associated with social trust and respect (see Table 5). This pattern suggests that nurses’ subjective risk perception, rather than self-reported exposure frequency, was more closely associated with their job esteem, consistent with the health belief model’s emphasis on the role of “perceived threat” in health-related attitudes (Ayasrah and Alrashdan, 2026). From a managerial perspective, while hospitals should strengthen actual protective measures, equal attention should be paid to reducing nurses’ risk perception through effective communication to avoid unnecessary panic and anxiety (Ayasrah and Alrashdan, 2026).

Another core finding of this study was that perceived hospital emphasis on occupational safety showed consistently positive associations with all five job esteem subscales (see Table 5). From the perspective of social exchange theory (Asher et al., 2025), nurses may reciprocate with greater professional commitment and a stronger professional identity when they perceive that their hospital invests resources in safety, improves protection systems, and demonstrates genuine concern for their well-being. An emphasis on safety is therefore not merely a means of preventing and controlling occupational risks but also a symbolic organizational signal that nurses’ lives and health are valued by the institution. Previous research has similarly shown that safety climate is significantly associated with nurses’ job satisfaction and intention to stay (Babapour et al., 2022; Yu et al., 2023). Hospitals should make the development of an occupational safety culture a strategic priority, rather than merely fulfilling regulatory compliance requirements.

## Limitations

Several limitations should be considered in this study. This study employed a cross-sectional design and therefore cannot establish temporal precedence or causal relationships. All data were self-reported, which introduces the possibility of reporting bias, including recall bias regarding the frequency and types of occupational injury or exposure, despite the use of anonymity and procedural measures to reduce common method variance. Recruitment relied on departmental nursing managers, and the hospital sample was selected using purposive sampling, which may have introduced selection effects and limited representativeness. The study was conducted only in Beijing, and the findings may not be fully generalizable to settings with different staffing structures, organizational climates, or healthcare systems. Occupational injury or exposure was assessed using study-specific items rather than a standardized scale, which may limit comparability with other studies. Finally, unmeasured confounding variables may have influenced the observed associations.

### Important practical implications for nursing management and occupational health promotion

This research has several practical implications for nursing management and occupational health promotion. Nursing managers may consider strategies such as optimizing staffing levels, conducting regular workload assessments, and improving the efficiency of daily routines to help address excessive occupational stress. At the same time, health promotion efforts should treat psychological risks with the same seriousness as physical hazards. Practical measures include establishing confidential reporting systems, offering post-event counseling, and implementing violence prevention strategies. Strengthening occupational protection and safety may be important for maintaining job esteem among nurses.

## Conclusion

In conclusion, this study found that nurses’ job esteem in Beijing was associated with occupational stress and occupational injury or exposure. Workload and time allocation and patient nursing care, were negatively associated with most dimensions of job esteem among nurses. The need for occupational safety and protection training and perceived hospital emphasis on occupational safety were positively associated with all dimensions of job esteem. Psychological harm and physical injury were negatively associated with most job esteem dimensions. Perceived risk of occupational exposure was associated with professional self-awareness, social trust and respect, professional competence, organizational respect and recognition, and professional authority and future value. These findings suggest that strategies aimed at reducing occupational stress, strengthening institutional safety support, and implementing targeted psychological and physical protection measures may be promising for supporting nurses’ job esteem and fostering a more supportive professional environment.

## Data Availability

The original contributions presented in the study are included in the article/supplementary material, further inquiries can be directed to the corresponding author.

## Footnotes

1
https://www.wjx.cn/
